# Transarterial chemoembolization combined with camrelizumab for recurrent hepatocellular carcinoma

**DOI:** 10.1186/s12885-022-09325-6

**Published:** 2022-03-14

**Authors:** Yusheng Guo, Yanqiao Ren, Lei Chen, Tao Sun, Weihua Zhang, Bo Sun, Licheng Zhu, Fu Xiong, Chuansheng Zheng

**Affiliations:** 1 Department of Radiology, Union Hospital, Tongji Medical College, Huazhong University of Science and Technology, Wuhan, 430022 China; 2 Hubei Province Key Laboratory of Molecular Imaging, Wuhan, 430022 China

**Keywords:** Hepatocellular carcinoma, Immune checkpoint inhibitors, TACE, Recurrence

## Abstract

**Purpose:**

To evaluate the efficacy and safety of transarterial chemoembolization (TACE) combined with camrelizumab (hereafter, TACE-camrelizumab) in the treatment of patients with recurrent hepatocellular carcinoma (R-HCC) after curative resection.

**Patients and methods:**

R-HCC patients who underwent TACE plus camrelizumab or TACE-alone from January 2016 to August 2021 were retrospectively evaluated. Patients were assessed for tumor response, progression-free survival, survival rates and adverse events.

**Results:**

Seventy-one patients were included in this study, including 20 patients in the TACE- camrelizumab group and 51 patients in the TACE-alone group. The objective response rate was 56.9% in the TACE-alone group and 40% in the TACE-camrelizumab group at 3 months (*P* = 0.201). The disease control rates were 84.3% in TACE-alone group and 80% in TACE-camrelizumab group at 3 months (*P* = 0.663). The progression-free survival (PFS) of the TACE-alone group was slightly longer than those of the TACE- camrelizumab group (9 months vs. 6 months). However, there were no statistically significant differences in the median PFS (*P* = 0.586). Similarly, there were no significant differences in the half-year and one-year survival rates (*P* = 0.304, *P* = 0.430). Multivariate analysis revealed that Neutrophil-to-lymphocyte ratio (NLR) was associated with PFS significantly. 75% patients developed at least one type of AEs related to camrelizumab in TACE-camrelizumab group, and no patients developed severe AEs.

**Conclusion:**

Comparing with TACE-Alone, the efficacy of TACE-camrelizumab for patients with R-HCC was similar. Meanwhile, the results of this study also indicated that TACE is still a better choice for patients with R-HCC.

## Introduction

Hepatocellular carcinoma (HCC) is the fifth most common carcinoma and the second leading cause of cancer-related death [1]. Although the recommended first-line treatments for patients with early-stage HCC have the potential to cure patients, 70% of patients have tumor recurrence within five years and only less than 30% of HCC patients can benefit from curative therapies [2, 3]. There have been no established treatments for recurrent HCC, although repeated liver resection remains one of the most effective choices for recurrent HCC treatment in suitable patients. However, due to many factors such as the limited reserve of liver function in the residual liver, postoperative adhesions, and multifocal recurrent tumors, re-resection is only suitable for a small number of patients [4–6].

Compared to surgical resection, transarterial chemoembolization (TACE), which combines targeted chemotherapy with ischemic necrosis caused by arterial embolization, is a well-tolerated procedure with limited liver toxicity and can be employed for any type of HCC [5, 7]. Therefore, TACE is widely applicable and practical in patients with intrahepatic HCC recurrence [8–10]. Despite that, it is reported that TACE did not have a better survival benefit than liver resection [11, 12]. Based on this, the question of whether TACE combined with other drugs can achieve better efficacy needs to be answered urgently.

In recent years, anti-PD-1 antibodies, including nivolumab, pembrolizumab, and camrelizumab, have demonstrated promising anti-tumor effects as monotherapy for HCC [13–16]. One study reported last year that nivolumab improved overall survival and progression-free survival in the patients with post-operative recurrence of malignant pleural mesothelioma, and it suggested immune checkpoint inhibitors were helpful for the patients with R-HCC [17]. But the negative results of single-agent ICIs in HCC hinted to us that combined therapy may gain better survival benefits [18, 19]. Fortunately, these kind of immune checkpoint inhibitors (ICIs) that reverse immune exhaustion have been shown to be effective in combination with TACE in the treatment of intermediate and advanced HCC [20]. According to reports, the principle of the combination therapy is that TACE increases tumor-specific CD8 + T cell response by killing HCC cells and causing the release of tumor-associated antigens [21], and increases programmed death receptor 1 (PD-1) and programmed death ligand 1 (PD-L1) expression in HCC [22, 23]. Recently, camrelizumab was approved in China as a second-line treatment for unresectable HCC. It showed high receptor occupancy on circulating T lymphocytes, high affinity for PD-1, and the different binding epitope from that of nivolumab and pembrolizumab [24, 25].

However, as far as we know, no studies have been reported on TACE combined with camrelizumab in R-HCC patients. Therefore, the objective of this study was to assess the efficacy and safety of TACE-camrelizumab verse TACE-alone in R-HCC patients.

## Material and methods

### Study design and patient selection

We reviewed the medical records of patients with recurrent HCC after curative resection who received treatment with TACE-camrelizumab or TACE-alone between January 2016 and August 2021 at Union Hospital, Tongji Medical College, Huazhong University of Science and Technology. This retrospective study was approved by the institutional review board of the Union Hospital, Tongji Medical College, Huazhong University of Science and Technology. Written informed consent for the patients’ data to be used for research purposes was obtained from all patients prior to treatment.

All of the patients included in this study met the inclusion criteria: (1) Adult patients whose diagnosis of HCC depended on the guidelines of the European Association for the Study of Liver and the American Association for the Study of Liver Disease [1]; (2) Patients were staged at BCLC-B or BCLC-C in accordance with the BCLC system; (3) Child–Pugh class A or B; (4) Eastern Cooperative Oncology Group (ECOG) performance status of 0–1. The exclusion criteria were: (1) Incomplete clinical information; (2) ECOG > 1; (3) Discontinuation of camrelizumab due to serious adverse events (AEs); (4) Loss to follow-up.

### TACE procedure

TACE was performed by placing a 5-F catheter (Cook, Bloomington, Indiana, USA) or a 2.7-F microcatheter (Progreat, Terumo, Tokyo, Japan) into hepatic tumor feeding arteries. Initially, an emulsion of 2–20 mL lipiodol and 20–60 mg epirubicin was administered into the target vessels under fluoroscopic guidance. Next, gelatin sponge particles (350–710 μm, Alicon, Hangzhou, China) were administered into the tumor donor arteries. Embolization was operated under fluoroscopic guidance until the stasis of arteries flow was occurred. Finally, reexamination angiography of the hepatic artery was performed to validate the devascularization.

### Camrelizumab administration

Patients were treated with camrelizumab within 2–3 weeks after TACE procedure. Camrelizumab was administered intravenously at a dose of 200 mg every 3 weeks. When serious adverse events (AEs) emerged, we reduced the dosage of drugs or interrupted it and symptomatic treatment such as glucocorticoids or immunosuppressant agents were administered, depending on the severity and the affected organs.

### Assessment of clinical outcomes and follow-up

All patients were followed up until 31 July 2021. Patients were evaluated every 6–8 weeks with laboratory and abdominal contrast-enhanced CT or MR. Tumor response was evaluated based on Response Evaluation Criteria in Solid Tumors (RECIST 1.1) and Modified RECIST (mRECIST) [26]. However, for consistency, only assessments using mRECIST were summarized in the following section. Follow-up imaging examinations at 3 months were compared with pretreatment imaging to determine objective response rate (ORR) and disease control rate (DCR).

The primary endpoint was progress-free survival (PFS). The secondary endpoints were ORR and DCR. PFS was defined as the period between the date of the initial TACE after being diagnosed with R-HCC and the date of the diagnosis of tumor progression or patient death. The ORR was defined as the percentage of patients with a complete response (CR) or partial response (PR). DCR was defined as the percentage of patients with CR, PR or stable disease (SD). Adverse events attributed to TACE or camrelizumab treatment were recorded and assessed by The Common Terminology Criteria for Adverse Events Version 5.0. In addition, postembolization syndrome, such as fever, pain, nausea and vomiting, is not considered an AE in itself, but rather an expected outcome of embolization therapy [27].

### Statistical analyses

All analyses were performed by using SPSS software, Version 24.0 (IBM, Armonk, New York). Discrete variables were represented by numbers with percentages and were calculated by Chi-square test, and continuous variables were presented as mean ± standard deviation and were calculated by Student’s t-test. Kaplan–Meier method and log-rank test were performed to evaluate the differences in PFS and survival rates between the two groups. The 95% confidence interval (CI) was calculated for median PFS and hazard ratio (HR). Log-rank test was used for univariate analysis, in which variables with *P* value less than 0.10 in univariate analysis were added to multivariate analysis. *P* < 0.05 indicated a statistically significance.

## Results

### Study population and patient characteristics

**Fig. 1:**
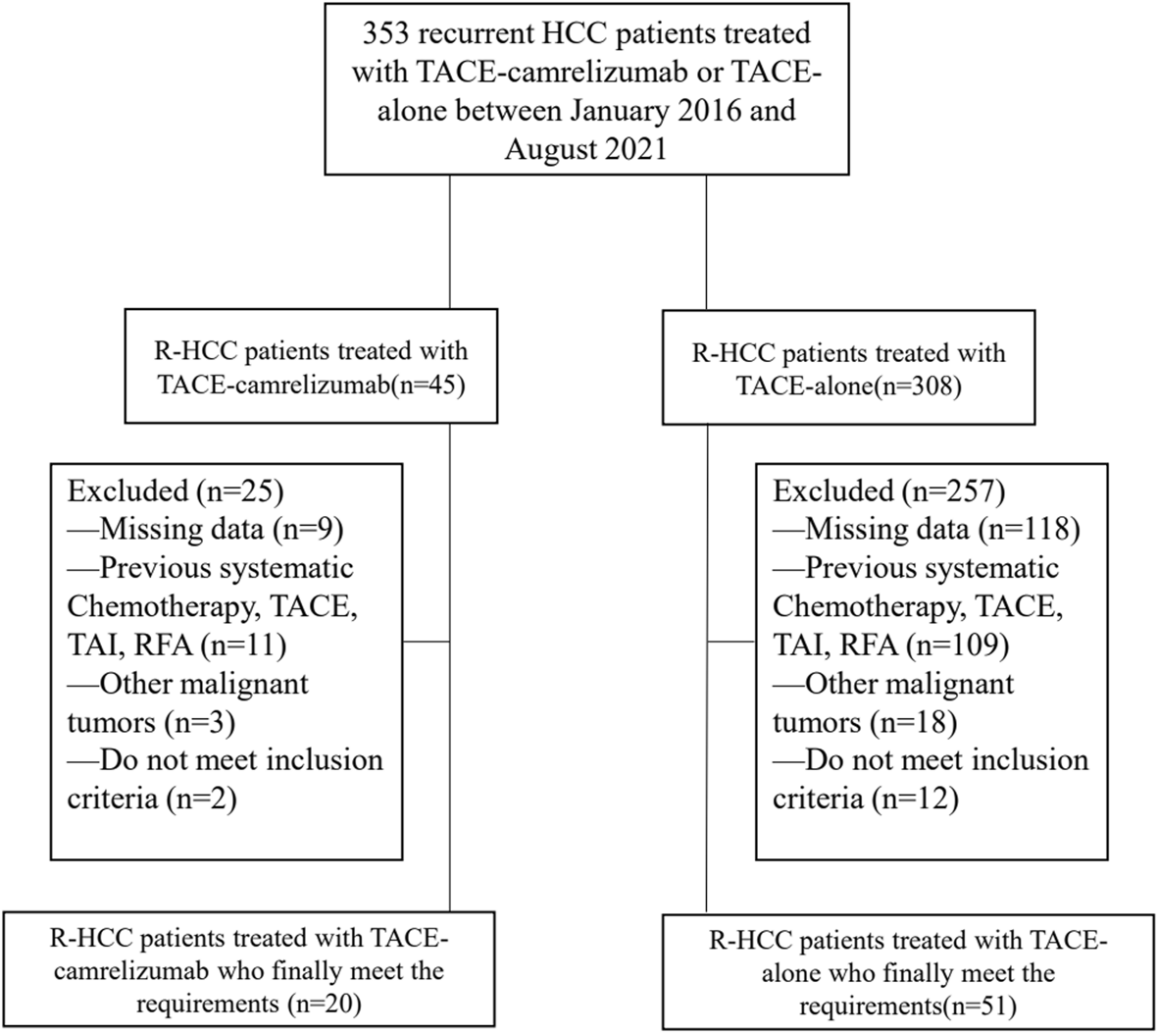
Flow chart shows the screening procedure for recurrent HCC patients with treated with TACE-camrelizumab or TACE-alone. HCC = hepatocellular carcinoma, R-HCC = recurrent hepatocellular carcinoma, TACE = transarterial chemoembolization, TAI = transcatheter arterial infusion, RFA = radiofrequency ablation

A total of 353 R-HCC patients underwent a treatment of either TACE- camrelizumab or TACE-alone in the study. 282 patients were excluded from the study and 71 patients were finally included in this analysis: 20 patients were treated with TACE-camrelizumab and 51 patients were treated with TACE-alone (Fig. 1). The baseline characteristics of the 71 patients were listed in Table 1. There was no significant difference in baseline characteristics between the two groups. The median follow-up period was 12 months (range, 4–55 months) in the TACE-alone group and 9 months (range, 5–15 months) in the TACE-camrelizumab group. In the TACE-alone group, 35 patients (68.6%) died at the end of follow-up, and in the TACE-camrelizumab group, 5 patients (25.0%) died.

**Table 1:** Baseline Characteristics

**Characteristics**	**TACE-alone group** (*N* = 51) (No, %; Mean ± SD)	**TACE-camrelizumab group** (*N* = 20) (No, %; Mean ± SD)	***P* value**
**Gender**			0.223
Male	43 (84.3%)	19 (95%)	
Female	8 (15.7%)	1 (5%)	
**Age (years)**	52.5 ± 10.7	50.3 ± 11.3	0.045
**ECOG** **performance**			0.769
0	21(41.2%)	9(45%)	
1	30(58.8%)	11(55%)	
**Hepatitis**			0.428
Hepatitis B	39 (76.5%)	17 (85%)	
Other	12 (23.5%)	3 (15%)	
**Child–Pugh class**			0.101
A	49 (96.1%)	17 (85%)	
B	2 (3.9%)	3 (15%)	
**BCLC stage**			0.113
B	14(27.5%)	2 (10%)	
C	37(72.5%)	18 (90%)	
**TB (µmol/L)**	19.08 ± 6.78	20.56 ± 10.6	0.486
**Albumin (g/L)**	37.3 ± 5.5	35.8 ± 3.00	0.064
**PT(s)**	14.1 ± 1.4	13.9 ± 1.1	0.600
**AST (µmol/L)**	39.2 ± 44.0	55.4 ± 23.2	0.124
**ALT (µmol/L)**	33.8 ± 21.0	39.5 ± 17.0	0.285
**PLR**	142.6 ± 75.9	157 ± 83.0	0.477
**NLR**	3.9 ± 1.8	3.5 ± 1.1	0.381
**Tumor size (cm)**	3.3 ± 2.3	4.1 ± 2.6	0.99
**Tumor number**			0.756
= 1	11 (21.6%)	5 (25%)	
≥ 2	40 (78.4%)	15(75%)	
**α-Fetoprotein** **level**			0.280
> 400 ng/mL	16(31.4%)	9(45%)	
≤ 400 ng/ml	35(68.6%)	11(55%)	
**Ascites**			0.254
Absent	44(86.3%)	15(75%)	
Present	7(13.7%)	5(25%)	
**Portal vein invasion**			0.531
Yes	14(27.5%)	7(35.0%)	
No	37(72.5%)	13(65.0%)	
**Extrahepatic metastasis**			0.994
Yes	28(54.9%)	11(55.0%)	
No	23(45.1%)	9(45.0%)	

*TACE*: Transcatheter arterial chemoembolization; *SD*: Standard deviation; *ECOG*: Eastern Cooperative Oncology Group; *TB*: Total bilirubin; *PT*: Prothrombin time; *AST*: Aspartate aminotransferase; *ALT*: Alanine aminotransferase; *BCLC*: Barcelona Clinic Liver Cancer; *PLR*: Platelet-to-lymphocyte ratio; *NLR*: Neutrophil-to-lymphocyte ratio

### Tumor response

The response of the tumor was determined by the abdominal contrast-enhanced CT or MR imaging. In the TACE–camrelizumab group, 1 patient (5%) achieved complete response, 7 patients (35%) achieved partial response, and 8 patients (40%) achieved stable disease. Therefore, the objective response rate and disease control rate were 40% and 80%, respectively. In the TACE–alone group, 18 patients (35.3%) achieved complete response, 11 patients (21.6%) achieved PR, and 14 patients (27.5%) achieved SD. Therefore, the objective response rate and disease control rate were 56.9% and 84.3%, respectively. The chi-square test indicated that there was no significant difference in objective response rate (*P* = 0.201) and disease control rate (*P* = 0.663) between the two groups.

### Progression-free survival and survival rates

**Fig. 2:**
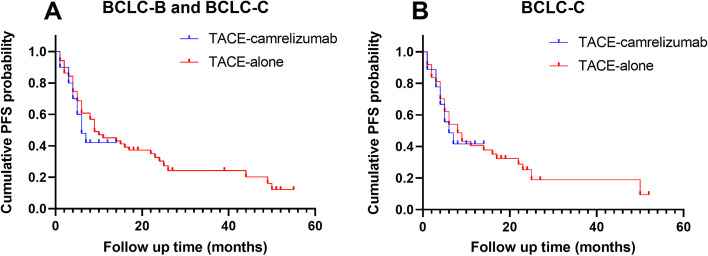
**A** Kaplan–Meier curves of cumulative PFS in recurrent HCC patients treated with TACE-camrelizumab or TACE-alone. HCC = hepatocellular carcinoma, PFS = progression-free survival. **B** Kaplan–Meier curves of cumulative PFS in recurrent HCC patients treated with TACE-camrelizumab or TACE-alone at the stage of BCLC-C. HCC = hepatocellular carcinoma, PFS = progression-free survival. BCLC = Barcelona Clinic Liver Cancer

The median PFS was 6 months (95%CI: 3.5, 8.6 months) in the TACE- camrelizumab group, and 9 months (95%CI: 3.0, 15.0 months) in the TACE-alone groups. No statistically significant difference was found between the two groups in the median PFS (*P* = 0.586) (Fig. 2A). In the patients at the stage of BCLC-C, the median PFS was 6 months (95%CI: 2.6, 9.4 months) in the TACE- camrelizumab group, and 8 months (95%CI: 4.6, 11.4 months) in the TACE- alone group with no significant difference (*P* = 0.862) (Fig. 2B). Univariate analysis indicated that BCLC stage, tumor number and Neutrophil-to-lymphocyte ratio (NLR) were associated with PFS (Table 2). Among these factors, multivariate analysis indicated that NLR was a prognostic factor for PFS (Table 3). The half-year survival rate was 95.0% in the TACE–camrelizumab group, and 86.3% in the TACE–alone group with no significant difference (*P* = 0.304). The one-year survival rate was estimated at 60.7% and 49.0% in the TACE–camrelizumab group and TACE–alone group, respectively (*P* = 0.430). In the patients at the stage of BCLC-C, the half-year survival rate was 94.4% in the TACE-camrelizumab group, and 83.8% in the TACE-alone group with no significant difference (*P* = 0.264). The one-year survival rate was 65.0% in the TACE- camrelizumab group, and 40.5% in the TACE–alone group with no significant difference (*P* = 0.177).

**Table 2:** Univariate analysis of prognostic factors for progression-free survival

Variables	HR (95% CI)	*P* value
**Gender**		
Male	Reference	
Female	1.634(0.793,3.368)	0.183
**ECOG performance**		
1	Reference	
0	0.846(0.485,1.474)	0.555
**Hepatitis**		
Hepatitis B	Reference	
Other	0.845(0.431,1.656)	0.624
**Child–Pugh score**		
B	Reference	
A	1.715(0.415,7.088)	0.456
**Age (years)**	0.987(0.961,1.015)	0.358
**AST (µmol/L)**	1.004 (0.997,1.010)	0.262
**ALT (µmol/L)**	1.006 (0.992, 1.020)	0.414
**PLR**	1.002(0.999, 1.006)	0.244
**NLR**	0.795(0.653, 0.969)	0.023
**Albumin (g/L)**	1.004 (0.913, 1.104)	0.934
**TB (µmol/L)**	0.973(0.933,1.015)	0.200
**PT (s)**	1.001(0.807,1.241)	0.991
**Tumor size**	0.979 (0.862, 1.112)	0.744
**BCLC stage**		
** C**	Reference	
** B**	0.557(0.299, 1.037)	0.065
**Tumor number**		
= 1	Reference	
≥ 2	0.551 (0.299, 1.017)	0.057
**α-Fetoprotein level**		
≥ 400 ng/mL	Reference	
< 400 ng/mL	1.168 (0.643, 2.121)	0.610
**Treatment method**		
TACE-alone	Reference	
TACE-camrelizumab	1.206(0.599,2.428)	0.600

Note. HR: Hazard ratio; CI: Confidence interval; ECOG: Eastern Cooperative Oncology Group; TB: Total bilirubin; PT: Prothrombin time; AST: Aspartate aminotransferase; ALT: Alanine aminotransferase; BCLC: Barcelona Clinic Liver Cancer; PLR: Platelet-to-lymphocyte ratio; NLR: Neutrophil-to-lymphocyte ratio

**Table 3:** Multivariate analysis of prognostic factors for progression-free survival

Variables	HR (95% CI)	*P* value
**BCLC stage**		
C	Reference	
B	0.551 (0.293, 1.035)	0.064
**Tumor number**		
= 1	Reference	
≥ 2	0.601 (0.325, 1.110)	0.104
**NLR**	0.802 (0.657, 0.980)	0.031

*BCLC*: Barcelona Clinic Liver Cancer; *NLR*: Neutrophil-to-lymphocyte ratio

### Adverse events

In the TACE-camrelizumab group, 9 patients (45%) developed fever (n = 7), abdominal pain (n = 4), nausea and vomiting (n = 4) within one week after TACE, and in the TACE-alone group, 16 patients (31.4%) developed fever (n = 11), abdominal pain (n = 8), nausea and vomiting (n = 6) within one week after TACE. After symptomatic treatment during hospitalization, the symptoms of all patients were alleviated or eliminated.

AEs related to camrelizumab are shown in Table 4. During the follow-up period, 15 (75%) patients developed at least one type of AEs after treatment with camrelizumab, and no patients developed severe AEs (more than grade 3). In addition, 1 patient (5%) developed pneumonitis, and the symptom was improved by glucocorticoids and interruption of camrelizumab. No treatment-related deaths occurred in this study.

**Table 4:** Adverse events related to camrelizumab administration in the TACE-Camrelizumab group

Adverse Event	All Events	CTCAE Grade		
		1	2	≥ 3
**RCCEP**	14 (70.0%)	10 (50.0%)	4 (20.0%)	0 (0%)
**Asthenia**	4 (20.0%)	4(20.0%)	0 (0%)	0 (0%)
**Rash**	4 (20.0%)	4 (20.0%)	0(0%)	0 (0%)
**Hypothyroidism**	2 (10.0%)	2 (10.0%)	0 (0%)	0 (0%)
**Pneumonitis**	1 (5.0%)	1 (5.0%)	0 (0%)	0 (0%)

*CTCAE* Common Terminology Criteria for Adverse Events, *RCCEP* Reactive cutaneous capillary endothelial proliferation

## Discussion

Because of the high risk of tumor recurrence after liver resection, it is urgent to find an effective treatment for the recurrence that can be used repeatedly and is suitable for patients with limited reserve of liver function. TACE is usually used in this kind of patients, but TACE alone cannot replace repeated liver resection. However, TACE increases tumor-specific CD8 + T cell response and programmed death receptor 1 (PD-1) and programmed death ligand 1 (PD-L1) expression, which provides an impetus for the exploration of immunotherapy after TACE [28]. In this study, we found that the efficacy of TACE-camrelizumab and TACE-alone is similar for R-HCC patients, with no significant statistical differences in PFS, objective response rate and disease control rate between the two groups. Previous study reported that NLR was useful prognostic factors in predicting outcomes in patients with HCC who underwent live resection, and multivariate analysis in our study found similar result, which suggested that inflammation played an important role in R-HCC [29, 30].

The median PFS in patients treated with TACE alone was 9 months (95%CI: 3.0, 15.0 months), longer than 6 months reported for TACE-camrelizumab. According to the previous research and our hypothesis, the addition of ICIs was helpful for patients who underwent TACE treatment in patients with primary HCC [23], but our study reports negative results both in PFS and survival rates. Similar negative results were reported in the study that evaluated the efficacy of TACE plus sorafenib [31], and one possible reason for this is the timing of sorafenib administration. More than half of the patients underwent sorafenib treatment 9 weeks after TACE, and yet the production of VEGF by triggering ischemic conditions might just exist for a short time [32], which reminded us that the condition of high expression of PD-1 and the increasing tumor-specific CD8 + T cell response in the tumor microenvironment might disappear soon. Another study comparing TACE plus sorafenib with TACE achieved positive results at median PFS (25.2 vs 13.5 months, *P* = 0.006). Besides the new criteria for progression, another possible factor of the positive results was that sorafenib was given 2–3 weeks before initial TACE was performed [33]. In this retrospective study, patients were treated with camrelizumab within 2–3 weeks after TACE procedure. It is possible that shrinking the interval between TACE and camrelizumab or giving the camrelizumab prior to TACE procedure after evaluating the condition of patients carefully can bring more survival benefits.

Some studies demonstrated the efficacy of transarterial embolization combined with ICIs in the treatment of primary HCC [20, 34]. However, recently, a study indicated that different immune therapy strategies should be considered for the treatment of primary HCC and recurrent HCC due to the significant differences in tumor microenvironments between the two groups [35]. According to the report, CD8 + T cells in recurrent tumors overexpressed KLRB1 (CD161) and displayed an innate-like low cytotoxic state, with low clonal expansion, unlike the classical exhausted state observed in primary HCC. Meanwhile, by compromising antigen presentation in DC cells and recruiting innate-like CD161 + CD8 + T cells, recurrent malignant cells could enhance immune evasion capacities and reduced cellular proliferation. As a result, the efficacy of ICIs was reduced in patients with R-HCC and normal in patients with primary HCC.

In the TACE-camrelizumab group, 70% of patients developed at least one type of AEs after treatment with camrelizumab, and all of the AEs were grade 1 or 2. These AEs were significantly improved after symptomatic treatment. Similar to the previous study, reactive cutaneous capillary endothelial proliferation (RCCEP) is the most common adverse event related to the camrelizumab, but RCCEPs were clinically controllable and self-limiting [24]. Overall, the treatment method of TACE–camrelizumab for R-HCC patients was safe and tolerable.

Our study had limitations. First, our study was retrospective, and the sample size in the two treatment groups was relatively small, therefore, it was difficult to make strong conclusions about these results. Second, our study was conducted in a single center. Finally, due to the relatively short period of follow-up time, this study did not achieve median OS. Therefore, it is necessary that prospective multicenter clinical trials be conducted in the future to validate our findings.

## Conclusion

In summary, this study is the first one to report the efficacy and safety of the TACE combined with camrelizumab in the treatment of R-HCC patients, and the results indicated that this combination therapy had a similar effect on R-HCC patients compared with TACE-alone, which reminded us that TACE was still a better choice for patients with R-HCC. At the same time, the treatment of TACE combined with camrelizumab had an acceptable safety profile, with no occurrence of severe AEs. Although this study did not demonstrate the superiority of TACE-camrelizumab over TACE-Alone, the results can definitely serve as an important reference for other research.

## Data Availability

The data analysed during this study are avaliable from the electrical medical database of Union Hospital, Tongji Medical college, Huazhong University of Science and Technology. Please contact the author Chuansheng Zheng (hqzcsxh@sina.com) upon reasonable requests.

## Abbreviations

AFP: Alpha-fetoprotein
BCLC: Barcelona Clinic Liver Cancer
CR: Complete response
CT: Computed tomography
EASL: European Association for the Study of the Liver
R-HCC: Recurrent hepatocellular carcinoma
MR: Magnetic resonance
RECIST: Response Evaluation Criteria in Solid Tumors
OS: Overall survival
PD: Progressive disease
PFS: Progressionfree survival
PR: Partial response
SD: Stable disease
TACE: Transarterial chemoembolization
RCCEP: Reactive cutaneous capillary endothelial proliferation
AEs: Adverse events
